# Evaluation of the MOVE online exercise programme for young people aged 13–30

**DOI:** 10.1007/s00520-023-07758-8

**Published:** 2023-06-05

**Authors:** Ellie Barnes, Gemma Hillier-Moses, Helen Murray, Clare Stevinson, Hester A. Franks, Lucy Gossage

**Affiliations:** 1 Queens Medical Centre, University of Nottingham School of Medicine, Nottingham, NG7 2UH UK; 2 MOVE Charity, 21 Cleeve Mount, Registered Charity Number 1165675, Loughborough, LE11 4SD UK; 3 School of Sport, Exercise and Health Sciences, Loughborough University, Loughborough, LE11 3TU UK; 4 Centre for Cancer Sciences, Translational Medical Sciences, Biodiscovery Institute, University of Nottingham, Nottingham, NG7 2RD UK; 5 Department of Oncology, Nottingham University Hospitals NHS Trust, Nottingham City Hospital, Hucknall Road, Nottingham, NG5 1PB UK

**Keywords:** Exercise, Cancer rehabilitation, Fatigue, Online intervention

## Abstract

**Purpose:**

To evaluate the MOVE exercise programme in supporting the recovery of young people affected by cancer.

**Methods:**

Participants in an 8-week exercise rehabilitation programme delivered online by cancer rehabilitation specialists completed self-reported questionnaires at baseline and after programme completion. Assessments included cancer-related fatigue (FACIT fatigue scale) and health-related quality of life (EORTC-QLC-30). Qualitative data were provided through written accounts of participant experiences and underwent content analysis.

**Results:**

Seventy-one participants commenced the exercise rehabilitation programme and 57 completed the programme and provided data for analysis (63% female; median age 22 years). Statistically significant improvements were observed in post-programme scores for all measured outcomes (cancer-related fatigue, quality of life, physical functioning, role functioning, emotional functioning). Content analysis of written experiences generated ten unique codes. The highest frequency codes were enjoyment (*n* = 34), motivation (*n* = 14) and fitness (*n* = 13).

**Conclusions:**

These findings indicate feasibility of delivery, acceptability to patients and physical and psychological benefits of a personalised online exercise rehabilitation programme for young people living with and beyond cancer. Further research involving a control arm and long-term follow-up would be beneficial.

**Implications for cancer survivors:**

These results support the inclusion of a personalised exercise programme as part of cancer rehabilitation for young people living with and beyond cancer.

## Introduction

Cancer survivorship has doubled in the UK over the last 40 years [1]. As a population, cancer survivors are at increased risk of several adverse health outcomes, including cancer-related fatigue (CRF), poor physical functioning, anxiety and depression [2]. Three quarters of young people living with and beyond cancer do not meet national exercise guidelines and are at a substantial risk of developing cardiovascular disease (CVD) in later life [3]. The James Lind Alliance Priority Setting Partnerships place well-being support for teenagers and young adults affected by cancer as a number one priority for research [4].

CRF is defined as a debilitating sense of physical and cognitive tiredness not proportional to activity or rest, associated with cancer and cancer treatment. It affects around half of cancer survivors during their recovery [2, 5–7]. Patients recovering from cancer treatment also face significant changes to their bodies and physical capabilities [8] and are at increased risk of developing CVD in later life; 40% of male and 28% of female early cancer survivors are classified in the CVD risk category [3]. Furthermore, young cancer survivors are more likely than individuals without cancer to have experienced a mental illness, a major depressive episode and suicidal thoughts [9]. Young survivors consistently report higher levels of distress than their peers, with insufficient social support, poor body image and fears of cancer recurrence influencing their well-being [10]. In addition, young people with cancer often experience a negative impact on their personal relationships [8]. Negative impacts on health-related quality of life (HRQoL) are also common with cancer survivors consistently scoring lower than matched groups; levels of CRF and emotional, social and role functioning remain severely affected well into survivorship [11].

Historically, people affected by cancer were advised against exercise in favour of rest during recovery. Now, it is clear that exercise following cancer treatment is not only safe and well-tolerated, with no increase in reported adverse effects compared to usual care, but is actually beneficial [12]. Increasing evidence also suggests physical activity and exercise can ameliorate the negative physical and psychological consequences of cancer treatment and may, for some cancers, reduce risk of recurrence [12–15].

Despite this encouraging evidence, less than half of cancer survivors are physically active [16]. Potential factors leading to physical inactivity are fear, lack of motivation, CRF, pain and a changed relationship with the body [3]. A study of 102 childhood and adolescent cancer survivors demonstrated that 75% of participants did not meet national exercise guidelines; the most common reason given was high levels of CRF [3]. Further qualitative research confirms side effects of treatment, self-motivation and time pressures as barriers to exercise participation and suggests anxiety surrounding physical abilities and acquisition of healthcare professional sign off to participate as additional obstacles [17]. An effective exercise intervention must therefore make efforts to overcome these common barriers.

Cancer rehabilitation aims to help people who have had cancer maintain and restore physical and emotional well-being by maximising the outcomes of their treatment and minimising the consequences of treatment and symptoms. Physical activity support and guidance is an important part of cancer rehabilitation. One example of an exercise intervention is provided by MOVE, a UK-based charity providing practical support to people affected by cancer to help them return to/start exercise [18]. MOVE offers an 8-week online exercise rehabilitation programme, designed and directed by level four cancer rehabilitation specialists (all of whom have completed an accredited level 4 personal training course in Cancer Rehabilitation and Exercise), to people living with and beyond cancer aged 13–30 years. Participants can self-refer to the programme via the MOVE website or can be referred by a healthcare professional (HCP) [18]. Each programme of activity is tailored specifically to the participant’s aims, health status and individual needs, and emailed to participants weekly. Participants are encouraged to complete pre- and post-programme questionnaires aimed at measuring CRF levels, perceived functioning and HRQoL. MOVE specialists follow up with the participants each week by telephone or video call to support and modify the programme as required.

This paper reports a service evaluation of MOVE charity’s online exercise rehabilitation programme. Changes pre- to post-programme in CRF, physical, emotional and role functioning, and HRQoL were examined and participant experiences were documented.

## Methods

### Participants

Eligible participants were aged 13–30 years with a previous or current diagnosis of cancer who completed the MOVE 8-week programme and provided informed consent for data collection. Participants who did not complete the MOVE 8-week programme and/or the pre- and post-participation questionnaires were excluded from analysis of the intervention. Non-completion rates and reasons for drop-out (where available) were documented.

### Ethical considerations

A service evaluation is a way to define or measure current practice within a service and is undertaken to benefit those who use a particular service. As a service evaluation, ethical approval is not required (Health Research Authority) [19]. All participants gave informed consent for their data to be used for the purposes of service evaluation and were notified of their right to withdraw at any time. Participants aged under 18 required the consent of their parent/legal guardian. Data was collected and stored in compliance with the General Data Protection Regulation (GDPR). Questionnaire results were anonymised prior to analysis using synthesised ID numbers and stored securely on an encrypted Excel workbook (Microsoft Corporation, Redmond, WA, USA) for access only by the lead researcher.

### Intervention

The programme is an 8-week exercise plan tailored to the individual needs and capabilities of the participant as well as weekly calls with a cancer rehabilitation specialist for support and adjustments. If a participant missed any of their weekly calls for any reason, the duration of the programme was extended to ensure that each participant received 8 weeks of tailored support, though this may be delivered over more than an 8-week period. The plan, delivered by email, includes details of a prescribed functional exercise for each day and periodic video guidance. Participants completed the prescribed exercises at home and are encouraged to keep activity diaries to reinforce habit building. Due to the personalised nature of the programme, time and intensity varies; however, every participant was prescribed at least 5 min of activity per day, 5–6 days a week. Exercises centre around aerobic and resistance training, but also include stability and flexibility-based exercise [18]. If a participant was unable to have a call with a cancer rehabilitation specialist for any reason, the duration of the programme was extended to ensure that each participant was able to access 8 support calls.

### Outcome measures

CRF was measured using the 13-item FACIT fatigue scale [20] (a license was obtained). The adult scale was selected since the majority of the sample were aged > 18 and the scale is very similar to the paediatric version (FACIT-F-PEDS) [21] (https://doi.org/10.1097/MPH.0b013e318095057a). Participants responded to each item on a 0–4 scale to indicate fatigue over the previous seven days with total scores ranging from 0 to 52 (with higher scores indicating less fatigue). The scale has good levels of validity and reliability demonstrated [22]. One question was omitted in error; this was treated in accordance with the FACIT policy on missing answers and taken into consideration during data interpretation.

HRQoL and functioning scores were measured using the European Organisation for Research and Treatment of Cancer QoL Questionnaire Core 30 (EORTC QLQ C-30, an academic user agreement was obtained) [23, 24]. Participants completed 29 questions regarding abilities, functioning and satisfaction in daily life over the previous 7 days (one question was omitted in error; this question [question 26 on EORTC QLQ-C30 version 3.0, “Has your physical condition or medical treatment interfered with your family life?]” is not relevant to the functional scales we assessed). The questions are split into functional scales; the scales physical functioning, role functioning, emotional functioning and HRQoL were chosen due to their relevance to MOVE’s objectives. All of the scales and single-item measures range in score from 0 to 100. For each scale, the raw score is calculated by estimating the average of the items that contribute to the scale and a linear transformation is used to standardise the raw score so that scores range from 0 to 100 (a higher score represents a higher (better) level of functioning). While this scale was developed for adults, it has some preliminary validation in children with cancer [25] and further work is ongoing (https://qol.eortc.org/questionnaire/aya/).

### Data collection

Two questionnaires were sent to participants by email using Google Forms: the first at baseline before starting the MOVE programme and the other after 8 weeks upon completing the programme. The questionnaires collected patient demographic information (sex, age, diagnosis, treatments received) and included both the FACIT fatigue scale and the QLQ C-30 QoL questions. The post-programme questionnaire also contained an opportunity for participants to provide a written account of their experience of the MOVE programme. Participant responses were uploaded to a Microsoft Excel spreadsheet by the lead researcher under synthesised ID numbers, without any identifiable information.

### Data analysis

Data were analysed using IBM SPSS Statistics software version 24 and GraphPad Prism V8.4.3. Summary scores for relevant outcomes were calculated in accordance with the FACIT and EORTC scoring manuals [26, 27]. Missing data (< 5%) were handled in accordance with scoring manuals by calculating mean sample value. Descriptive statistics were calculated to establish baseline characteristics of the sample. Wilcoxon’s signed rank testing was used for statistical analysis of FACIT and EORTC QLQ C-30 scores [8]. Participants meeting the minimum important difference, the smallest change in a patient-reported outcome that patients and clinicians would perceive as important, were calculated for fatigue scores [28]. Subgroup analysis compared outcomes between participants with haematological cancers and solid cancers using the Mann-Whitney *U* test. On all graphs * = *p* < 0.05, ** = *p* < 0.005, *** = *p* < 0.001.

Participants’ written experiences were collected into a separate Microsoft Excel database and underwent qualitative content analysis by EB [29]. Content analysis is a systematic method of distilling qualitative data into related categories that allow inferences to be drawn. Codes were established by examining the answers for common patterns surrounding their experience with the programme in line with Bengtsson et al.’s guidance on content analysis [29]. The codes were defined according to the lead researcher’s interpretation of participants’ meaning, with direct quotes extracted to represent each code. Frequencies of the codes in the text were then determined.

## Results

### Participant characteristics

**Fig. 1:**
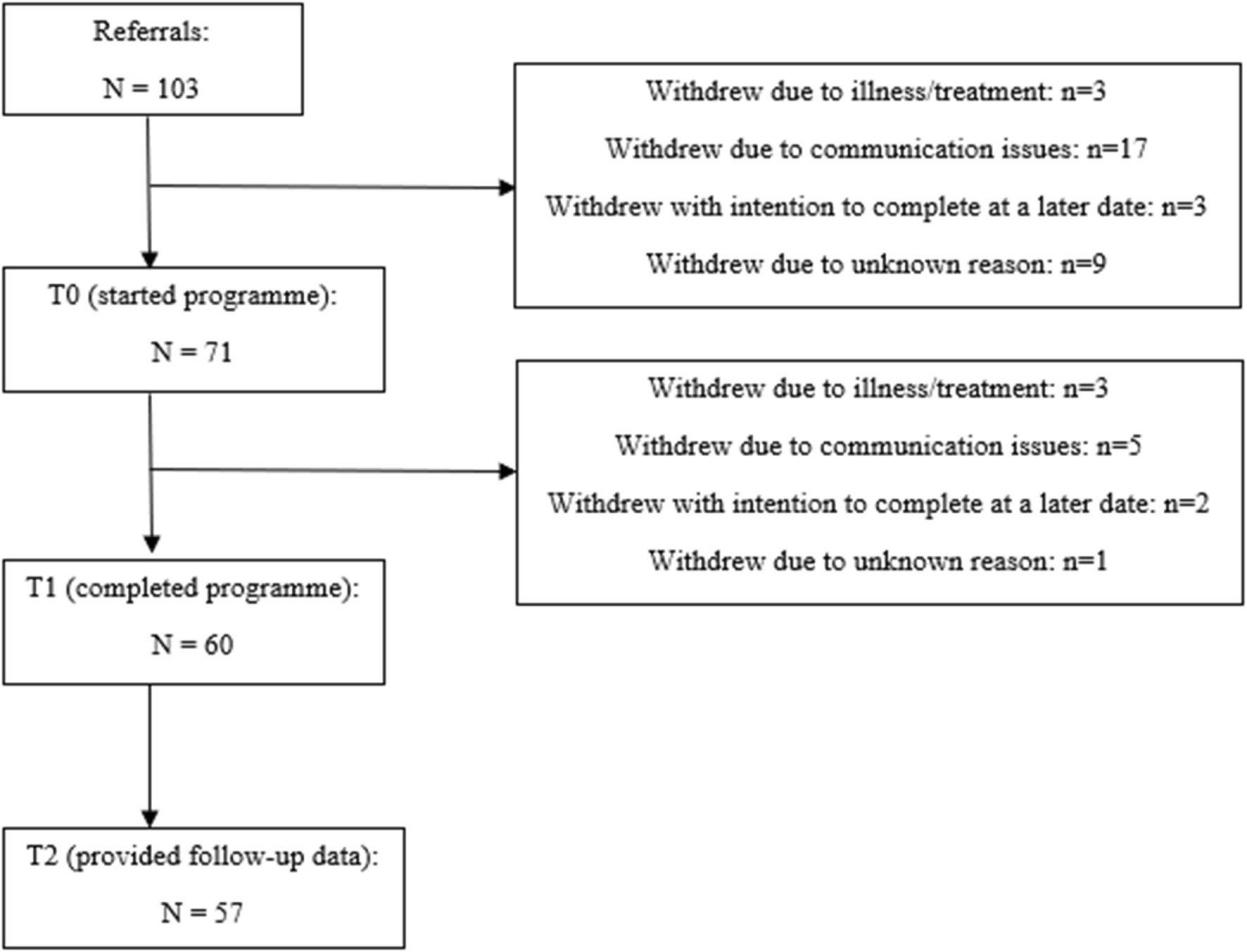
Flowchart of referrals. Flow of referrals indicating uptake of MOVE programme from referral, completion and inclusion in analysis

Between November 2018 and July 2020, 103 young people were referred to MOVE. Out of these, 71 started the online programme, 60 completed the programme (i.e. completed 8 calls with their cancer rehabilitation specialist) and 57 completed the post-programme questionnaire and were included in this analysis (Fig. 1). Once started, the completion rate of the exercise programme was 84.5%.

Participants were included in the final analysis if they completed the exercise programme and the post-programme questionnaire, a total of 57 participants. Demographic and treatment-related characteristics are shown in Table 1. The median age of participants included in the final analysis was 22 with more females than males. Haematological cancers were more common than solid tumours (59.6 vs 40.4% respectively) with the most common diagnosis being Hodgkin’s Lymphoma. The majority of participants had undergone chemotherapy (80.7%). Most participants had finished cancer treatment at the time of participation, with 73.7% having no current evidence of cancer while 8.8% were living with cancer.

**Table 1:** Demographic and treatment-related characteristics of participants completing the programme

			Number (%)
Number of participants completing exercise programme and post-programme questionnaire			57 (100)
Age	Median (years)		22
	13–18 years		11 (19.3)
	19–24 years		35 (61.4)
	25–30 years		11 (19.3)
Sex	Male		21 (36.7)
	Female		36( 63.2)
Diagnosis	Haematological malignancy		34 (59.6)
		Hodgkin’s Lymphoma	21 (36.8)
		ALL	6 (10.5)
		Non-Hodgkin’s Lymphoma	3 (5.3)
		Aplastic anaemia	2 (3.5)
		Burkitt Lymphoma	1 (1.8)
		AML	1 (1.8)
	Solid tumour		23 (40.4)
		Brain tumour	9 (15.8)
		Testicular cancer	3 (5.3)
		Sarcoma	3 (5.3)
		Breast cancer	2 (3.5)
		Thyroid cancer	2 (3.5)
		Other^a^	4 (7.0)
Treatment modalities^b^	Chemotherapy		46 (80.7)
	Radiotherapy		21 (36.8)
	Surgery		26 (45.6)
	Immunotherapy		8 (14.0)
	Hormone therapy		6 (10.5)
	Stem cell transplant		4 (7.0)
Number of treatment modalities	1		24 (42.1)
	2 or more		33 (57.9)
Current cancer status at the time of participation	Finished treatment with no evidence of cancer		42 (73.7)
	Undergoing active treatment		10 (17.5)
	Living with cancer but not currently receiving treatment		5 (8.8)

^a^Other = 1 participant with each of cervical, salivary gland, colon and melanoma

^b^*n* > 57 and % > 100 as many participants had more than one treatment modality

### Impact of MOVE programme

**Fig. 2:**
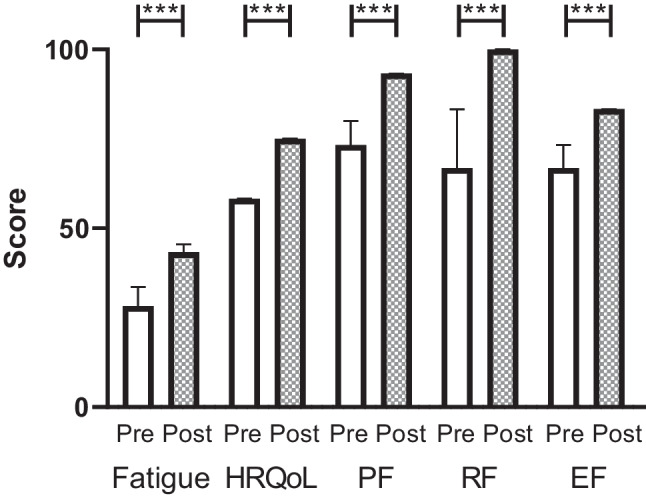
Impact of the MOVE programme on analysed outcome measures. Pre-programme (Pre, open bars) and post-programme (Post, hashed bars) scores (median, 95% confidence interval) are shown for cancer-related fatigue (Fatigue), health-related quality of life (HRQoL), physical functioning (PF), role functioning (RF) and emotional functioning (EF) as assessed by FACIT and EORTC QLQ C-30 scales. Maximum score for all outcomes was 100 except for cancer-related fatigue which scored out of a maximum of 52. ***p < 0.001 by Wilcoxon’s matched pairs signed rank test

Five outcomes were measured via the questionnaires completed by participants: CRF, physical functioning, role functioning, emotional functioning and QoL. In all five, there was a statistically significant increase in scores in the post-programme questionnaire, indicating an improvement (all *p* < 0.001, Fig. 2). The biggest difference was observed in CRF scores with a baseline/pre-programme median of 28.2 (IQR 13) out of a maximum of 52 and a post-intervention median of 43.3 (IQR = 9.8), followed by HRQoL (pre-programme median 58.3 out of a maximum of 100 (IQR 26.8), post-programme median 75 (IQR 25)). The minimum important difference for the fatigue scale (MID = 3) was observed in 43 participants (75.4%).

### Subgroup analysis

**Fig. 3:**
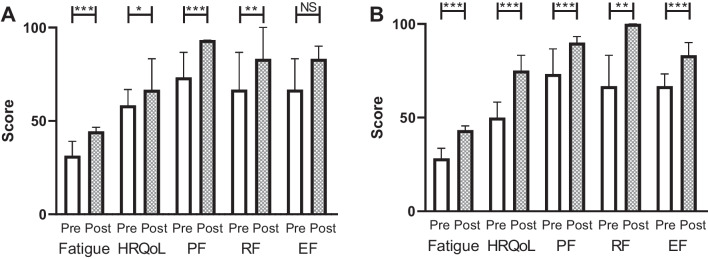
Subgroup analysis in solid tumour and haematological cancer participants. Pre-programme (Pre, open bars) and post-programme (Post, hashed bars) scores (median, 95% confidence interval) are shown for cancer-related fatigue (Fatigue), health-related quality of life (HRQoL), physical functioning (PF), role functioning (RF) and emotional functioning (EF)) as assessed by FACIT and EORTC QLQ C-30 scales. Maximum score for all outcomes was 100 except for cancer-related fatigue which scored out of a maximum of 52. **A** Solid tumour participants. **B** Haematological cancer participants. Wilcoxon’s matched pairs signed rank test *p < 0.05, **p < 0.005, ***p < 0.001

Treatment modalities, treatment teams and outcomes often differ between solid tumours and haematological cancers, and standard supportive services are distinct. We therefore carried out a subgroup analysis to establish whether the overall improvements in post-programme scores were evident in both haematological and solid tumours. In both subgroups, statistically significant improvements were still seen in all outcomes, except for emotional function in solid tumour participants (Fig. 3A and B). While the magnitude of the improvements was numerically greater in haematological cancer participants, this was not statistically significant for any of the outcome measures (Mann–Whitney *U* test, data not shown).

### Content analysis

Forty-nine participants provided a written account of their experience of the MOVE online programme (eight participants declined). Content analysis of participants’ written experiences established 10 codes (Table 2). The code with the highest frequency among participant responses was general enjoyment, with 34 participants expressing joy at completion of the programme, for example: “I’ve had so much fun I can’t believe it’s over” (Table 2). The next highest frequency codes were motivation (*n* = 14) and fitness (*n* = 13). One participant expressed that the programme has changed her life: “MOVE has changed my life and challenged me, when I was diagnosed with cancer it took so much away from me including my confidence. MOVE has enabled me to be confident and know that eventually I can get back to where I was and even better”. The highest frequency codes were enjoyment (*n* = 34), motivation (*n* = 14) and fitness (*n* = 13).

**Table 2:** Content analysis findings. Codes found in participants’ written responses, definitions according to researcher’s interpretation of participants’ meaning, example quotes taken from the responses and their frequency among the responses

Code	Description	Example quotes	Count
General enjoyment	Expresses joy/having had fun	“it was amazing”; “I've had a great time”; “I loved it”	34
Motivation	Feeling motivated/encouraged/inspired	“given me the motivation I needed”;	14
Fitness	Expresses improvement in endurance/strength/performance	“build back my basic strength”; “become more physically fit”	13
Individual needs	Appreciation for personalised nature of programme	“perfect for me”; “tailored exactly to what I needed”	13
Support	Feeling supported by MOVE team	“so supportive”; “the support was amazing”	12
Mood	Feeling more positive/happier, feeling less down/anxious	“allowed me to remain positive”; “release anxieties”	10
Confidence	Increase in confidence in abilities/body, self esteem	“my confidence has grown massively”; “makes me feel better about myself”	8
Function	Increase/improvement in daily activities whether work or social	“engage in more activity”; “really helpful with my day to day activities”	8
Energy	Reduction in fatigue/noticeable increase in energy	“new burst of energy”; “I feel energised and alive”	7
Health	Increase in general health/decrease in general symptom burden	“I have seen an improvement in my health”	2

Of the 49 responses, 4 contained a “negative” comment. Two of these comments detailed persistent CRF despite completion of the programme. The other two were constructive criticism of the online service used and expression of a need for reminders before follow-up phone calls.

## Discussion

To our knowledge, this is the first study to evaluate an internet-based, tailored exercise intervention specifically aimed at young people living with and beyond cancer. The high completion rates after starting the programme of 84.5% show that a personalised, online exercise rehabilitation programme for young people living with and beyond cancer is feasible to deliver and acceptable to patients. The principal aim of this evaluation was to examine the impact of the MOVE exercise programme on participants’ fatigue and quality of life. Our results demonstrate a statistically significant improvement in all outcome measures after completion of the programme; participants reported higher QoL, physical, emotional and role functioning and lower CRF after completion. This is strengthened by the qualitative data, which illustrates participants’ motivation, feelings of support and increased fitness. The prospective approach to data collection facilitated the analysis of multiple outcomes and minimised the role of preconceived biases.

Several previous studies have reported exercise interventions as markedly less effective in haematological cancers, with suggestion that the higher prevalence of anaemia, cachexia and psychological distress among this group are barriers to participants’ outcomes [13, 30]. In contrast to this, we saw benefit in both solid and haematological cancers, and numerically greater improvements in scores in haematological cancers (although not a statistically significant difference in benefit). This study therefore demonstrates that exercise intervention is beneficial in both haematological and solid cancers. Further analysis is needed to examine whether there is a difference between participants self-referring and those referred by a healthcare professional.

For those participants who had finished treatment, it would have been helpful to capture data on the time gap between finishing treatment and starting the programme; a longer gap would indicate the improvements seen after completion of the programme are more likely due to the programme rather than natural improvement over time. Furthermore, we accept that including a comparison group that received usual care would add further weight to our conclusions. Including a “usual care group” could distinguish between natural improvement over time and improvement due to the intervention. Consideration should be given to a case-control study of the MOVE programme to demonstrate the benefits more conclusively along with long-term follow-up after completion of the programme to establish the sustainability of benefit. Looking to existing evidence, the benefits of exercise interventions for cancer survivors can persist for up to 5 years following the intervention; however, they rely on participants maintaining a healthy, active lifestyle [2, 3]. MOVE addresses this with habit building, reinforced by the activity diaries, and patient education.

One of the 13 questions from the FACIT fatigue scale was accidentally omitted from the questionnaire during the design process (“I am too tired to eat”) and thus not answered by the participants. While CRF scores were still able to be calculated using the policy for treating missing answers, the absence of this question compromises the accuracy of assessment of participants’ CRF levels [4, 5, 23, 24]. This omission is consistent in both the pre- and post-programme questionnaires.

These results confirm similar findings from studies examining the role exercise intervention plays in cancer recovery [13, 17, 32]. One such study evaluated the RENEW programme, a 12-week exercise programme delivered by level 4 cancer rehabilitation specialists for young adult cancer survivors. Also based in the UK, this programme bears many similarities to MOVE, the key difference being in MOVE’s internet-based delivery. They concluded the programme had a positive impact on participants’ recovery, evidenced by significant improvements seen in physical function, CRF and HRQoL measures [6]. One can also see such effects reflected in the results of systematic reviews and meta-analyses [13, 32].

The content analysis of participants’ written experiences suggests the programme was successful in motivating participants, and, owing to its personalisation and virtual delivery, MOVE addresses lack of time and resources, common barriers in achieving programme completion [33, 34]. The weekly regimens aid in building habits, while telephone/video calls with a specialist provide social support and accountability. This is reflected in the completion rate of the MOVE programme with 84.5% of participants completing the 8-week programme (though some participants completed it over a longer than 8-week period). This compares favourably with a similar intervention, the Trekstock programme, at 50% of participants [32]. However, a number of participants did not complete the programme and did not respond to multiple attempts to contact them. Understanding reasons for programme discontinuation is an important challenge in undertaking service evaluations and should be a focus for future research.

The use of internet-based interventions for cancer rehabilitation is a relatively new and unexplored area. It is possible that the lack of in-person contact provides an easier route to non-completion for participants since a key factor in achieving adherence is supervision [34]. However, the flexibility of an online approach allows for a more seamless integration of the programme into everyday life and offers people control over their own rehabilitation journey. This is also a population of people for whom travelling to gyms may not be an option. Studies evaluating the acceptability of online interventions for cancer survivors, although limited, highlight the time- and cost-effectiveness of such an approach [35, 36].

The virtual roots of the programme and its accessibility are increasingly relevant; the unique position of MOVE during the COVID-19 pandemic facilitated the continuation of the programme amidst delays or cancellations of other cancer support services. It is likely that online approaches will increasingly become integrated into healthcare [37]. While the online nature of the MOVE programme enabled it to keep running during the pandemic, the prolonged periods of isolation imposed due to national lockdown and shielding requirements may have negatively affected HRQoL measures. Conversely, a climate of increased mental health awareness coupled with a nation-wide emphasis on home exercise is complementary to the values of the MOVE programme and so could have counteracted this.

The results of this evaluation may not be generalisable to all cancer patients. A larger sample size, with subgroup analysis according to treatment history, current treatment status and cancer type and stage would further validate these results. Different treatment options and participants’ treatment status, for instance, will influence outcome measures and completion rates [5, 6, 11]. A more detailed analysis of barriers to participation is also required, in particular socio-economic status and access to the technology required to participate, e.g. smartphone/computer access.

It is interesting that more females than males participated in the programme. There is some evidence that women in general may be more willing to participate in an exercise-based cancer rehabilitation programme than men [38, 39]. It is possible that there is also a referral bias in that healthcare professionals perceive females as more likely to engage with a programme. It is not surprising that the most common diagnosis was Hodgkin’s Lymphoma as lymphomas are the most common group of cancers in young people. However, the participants included in this analysis were enrolled at an early stage of MOVE and referrals were received from a smaller number of centres than they are currently and the expertise of the professionals referring into the service may to some extent account for this finding.

This study also aimed to provide insight into the potential for future growth and development of the MOVE programme. Some proposals for improvements that emerged in participants’ written experiences included the addition of reminder texts prior to a phone call (which the programme has since incorporated) and consideration for ongoing peer support. Since the completion of this study, some of the young people that have completed the MOVE programme have created their own online support group to allow young people affected by cancer to continue to support each other as they live with or beyond cancer.

### Strengths and limitations

This is a service evaluation of an online exercise rehabilitation programme delivered to young people aged 13–30 years with a previous or current diagnosis of cancer. As such, the results of this evaluation may not be generalisable to all cancer patients. We acknowledge that one question was missing from the FACIT questionnaire and that further evidence could have been gained from focus groups and semi-structured interview. Future research involving longer follow-up and greater numbers would be helpful.

## Conclusions

In summary, the evaluation findings demonstrated significant improvements in physical and psychological well-being following an online cancer rehabilitation programme in young cancer survivors.

These improvements in patient outcomes coupled with the engagement in the programme evidenced by the qualitative data confirm the programme’s role in cancer rehabilitation and support further growth and development [9, 13]. We would strongly advocate further research to understand the impact of cancer rehabilitation programmes and the barriers to participation in such programmes, in order to facilitate more widespread funding of such initiatives.

## Data Availability

Selected data fields are available by contacting the corresponding authors.
